# Haploinsufficiency for *ANKRD11*-flanking genes makes the difference between KBG and 16q24.3 microdeletion syndromes: 12 new cases

**DOI:** 10.1038/ejhg.2017.49

**Published:** 2017-04-19

**Authors:** Francesca Novara, Berardo Rinaldi, Sanjay M Sisodiya, Antonietta Coppola, Sabrina Giglio, Franco Stanzial, Francesco Benedicenti, Alan Donaldson, Joris Andrieux, Rachel Stapleton, Astrid Weber, Paolo Reho, Conny van Ravenswaaij-Arts, Wilhelmina S Kerstjens-Frederikse, Joris Robert Vermeesch, Koenraad Devriendt, Carlos A Bacino, Andrée Delahaye, S M Maas, Achille Iolascon, Orsetta Zuffardi

**Affiliations:** 1 Department of Molecular Medicine, University of Pavia, Pavia, Italy; 2 Department of Clinical and Experimental Epilepsy, NIHR University College London Hospitals Biomedical Research Centre, UCL Institute of Neurology, London, UK; 3 The Epilepsy Society, Chalfont-St-Peter, Bucks, UK; 4 Epilepsy Centre, Department of Neuroscience, Reproductive and Odontostomatological Sciences, Federico II University, Naples, Italy; 5 Medical Genetics Unit, Department of Clinical and Experimental Biomedical Sciences ‘Mario Serio’, University of Florence, Florence, Italy; 6 Department of Biomedical Experimental and Clinical Sciences ‘Mario Serio’, Medical Genetics Unit, Meyer Children's University Hospital, Florence, Italy; 7 Servizio di Consulenza Genetica, Centro Provinciale di Coordinamento della Rete delle Malattie Rare, Azienda Sanitaria dell'Alto-Adige, Bolzano, Italy; 8 Department of Clinical Genetics, St Michael’s Hospital, Bristol, UK; 9 Institut de Génétique Médicale, Hôpital Jeanne de Flandre, CHRU de Lille, France; 10 Genetic Health Service NZ-Northern Hub, Building 30 Auckland City Hospital, Auckland, New Zealand; 11 Merseyside and Cheshire Clinical Genetics Service, Liverpool Women’s (NHS) Foundation Hospital Trust, Liverpool, UK; 12 Department of Genetics, University of Groningen, University Medical Center Groningen, Groningen, The Netherlands; 13 Department of Genetics, University of Groningen, University Medical Center Groningen, Groningen, The Netherlands; 14 Laboratory of Cytogenetics and Genome Research, Center of Human Genetics, KU Leuven, Leuven, Belgium; 15 Department of Molecular and Human Genetics, Baylor College of Medicine, Houston, TX, USA; 16 Texas Children's Hospital, Houston, TX, USA; 17 INSERM, UMR 1141, Robert Debré University Hospital, Paris, France; 18 Cytogenetics Unit, AP-HP, Jean Verdier Hospital, Bondy, France; 19 Paris 13 University, Sorbonne Paris Cité, UFR SMBH, Bobigny, France; 20 Department of Pediatrics, Academic Medical Center, Amsterdam, The Netherlands; 21 Department of Clinical Genetics, Academic Medical Center, Amsterdam, The Netherlands; 22 Dipartimento di Medicina Molecolare e Biotecnologie Mediche, Università degli Studi di Napoli Federico II, Naples, Italy; 23 CEINGE Biotecnologie Avanzate Scarl, Naples, Italy

## Abstract

16q24 deletion involving the *ANKRD11* gene, ranging from 137 kb to 2 Mb, have been associated with a microdeletion syndrome characterized by variable cognitive impairment, autism spectrum disorder, facial dysmorphisms with dental anomalies, brain abnormalities essentially affecting the corpus callosum and short stature. On the other hand, patients carrying either deletions encompassing solely *ANKRD11* or its loss-of-function variants were reported in association with the KBG syndrome, characterized by a very similar phenotype, including mild-to-moderate intellectual disability, short stature and macrodontia of upper incisors, with inter and intrafamilial variability. To assess whether the haploinsufficiency of *ANKRD11*-flanking genes, such as *ZFPM1*, *CDH15* and *ZNF778*, contributed to either the severity of the neurological impairment or was associated with other clinical features, we collected 12 new cases with a 16q24.2q24.3 deletion (*de novo* in 11 cases), ranging from 343 kb to 2.3 Mb. In 11 of them, the deletion involved the *ANKRD11* gene, whereas in 1 case only flanking genes upstream to it were deleted. By comparing the clinical and genetic features of our patients with those previously reported, we show that the severity of the neurological phenotype and the frequency of congenital heart defects characterize the deletions that, besides *ANKRD11*, contain *ZFPM1*, *CDH15* and *ZNF778* as well. Moreover, the presence of thrombocytopenia and astigmatism should be taken into account to distinguish between 16q24 microdeletion syndrome and KBG syndrome. The single patient not deleted for *ANKRD11*, whose phenotype is characterized by milder psychomotor delay, cardiac congenital malformation, thrombocytopenia and astigmatism, confirms all this data.

## Introduction

KBG syndrome is one of many rare diseases associated with intellectual disability but with otherwise nonspecific clinical handles. The presence of macrodontia of the permanent maxillary incisors remains the most evocative clinical sign. Other common features suggested as major criteria for the clinical diagnosis are: characteristic facial dysmorphisms, hand anomalies, neurological involvement, delayed bone age, costovertebral anomalies, postnatal short stature and the presence of a first-degree relative with KBG.^1^

Heterozygous loss-of-function (LoF) variants in the *ANKRD11* gene at 16q24.3 have been identified to be causative for KBG syndrome.^2, 3^ Mild-to-moderate intellectual disability is a KBG feature but individuals with only minor learning difficulties have been reported, so that not only *de novo* but also inherited or familial cases have been observed.^3^ Vertical transmission has been related to low-grade mosaicism as well, responsible for a milder phenotypic presentation.^4, 5^ Beside single-nucleotide variants,^2, 3, 6, 7, 8, 9, 10^ KBG syndrome has been associated with 16q24.3 microdeletions or intragenic microduplications encompassing only the *ANKRD11* gene and thus resulting in its haploinsufficiency (Supplementary Table S1).^4, 5, 11, 12, 13, 14^ On the other hand, at least eight cases of 16q24.3 microdeletions involving other genes besides *ANKRD11* have been reported so far (Supplementary Table S1),^3, 15, 16, 17^ and, among the flanking genes, *ZNF778*, *CDH15*, *ZFPM1* and *SPG7* have been pointed out for a possible role in the resulting phenotype.^16^ By comparing clinical features of patients with *ANKRD11* LoF variants *versus* those with 16q24.3 microdeletions, Ockeloen *et al*^3^ concluded that the frequency of congenital anomalies, seizures and behavioral problems appeared to be similar in both groups. However, owing to the low number of patients, the authors could not exclude that the severity of neurological symptoms and intellectual disability was greater in cases where the deletion included other genes, besides *ANKRD11*.

We report on 12 new individuals with deletions of 16q24.2q24.3, originated *de novo* in 11 patients and of unknown origin in the remaining one. We compare the clinical and genetic features of our patients with those previously reported, in order to clarify whether haploinsufficiency of *ANKRD11*-flanking genes contributed to either the severity of the phenotype or the presence of distinctive clinical features.

## Patients

Written informed consent was obtained from patients’ parents or patients themselves. Only some have given consent for picture publication. The relevant institutional ethics committees approved this study. All patient data have been submitted to ClinVar (https://www.ncbi.nlm.nih.gov/clinvar/).

### Patient 1 (ClinVar SCV000328241)

**Figure 1:**
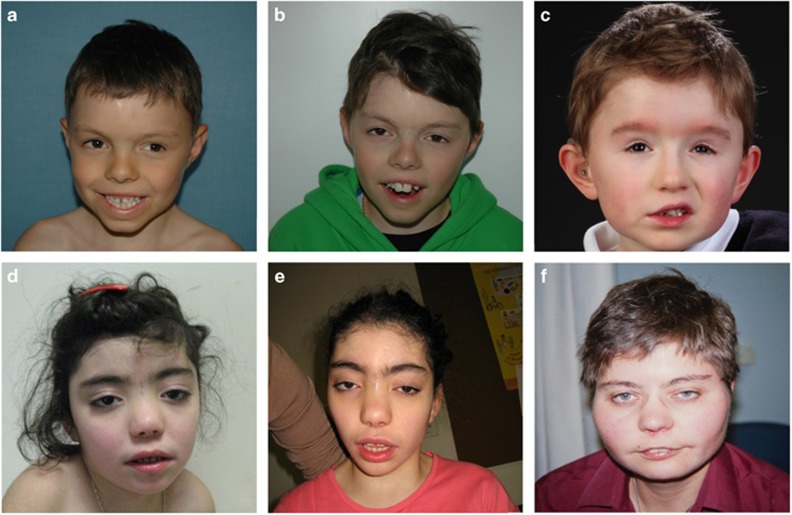
Facial pictures of four new patients: patient 1 at 8 and 11 years of age (**a**, **b**), patient 5 at 6 years of age (**c**), patient 11 at 7 and 14 years of age (**d**, **e**), patient 12 at 13 years of age (**f**).

He is the second child of unrelated and healthy parents, born at 41+3 week of gestation (WG) with normal auxological parameters. At birth a ventricular septal defect (VSD) and left cryptorchidism were observed. Psychomotor development was slightly delayed and from the infancy he presented moderate–severe astigmatism as well. At the age of 5 years, short stature was evident. When he was 6 years and 11 months old, a psychometric evaluation evidenced a borderline intellectual disability with overall IQ of 72 and main difficulties in language and motor skills. Learning disabilities and attention deficit-hyperactivity disorder were also diagnosed. At 11 years and 9 months, his stature was 128 cm (< −2 SD). At the age of 12 years and 3 months, X-ray revealed bone age delay and bilateral prominent C7 transverse processes. Facial dysmorphisms included: prominent forehead, thin hair in the temporal regions, synophrys with sparse eyebrows, prominent and posteriorly rotated ears, high nasal bridge and nose with large root, anteverted nostrils with thickened alae of the wings. Face was slightly asymmetric with mild retrogenia (Figures 1a and b). Mouth was maintained preferentially open, with trapezoidal morphology, full lips and downturned corners of the mouth. Macrodontia of upper central incisors was evident. Moderate brachydactyly was noted together with severe clinodactyly and shortness of fifth fingers, proximally placed thumbs and flat feet. Platelets count was normal.

### Patient 2 (DECIPHER patient SMB255327, ClinVar SCV000328242)

He was born at 38 WG following a normal pregnancy. Birth weight was ~2 kg (<−2 SD) and he experencied feeding problems for the first 6 months. Neonatal investigations revealed a VSD. Motor milestones were delayed, and hypotonia was present from the age of 1 year. He had a diagnosis of cerebral palsy. An abnormality of his primary dentition, not otherwise defined, was noted but adult teeth were normal. At the age of 22 years old, verbal IQ was 67 and performance IQ of 62. On the examination at the age of 26 years old, occipito-frontal circumference (OFC) was >+1 SD, weight was normal, height<−1 SD and body mass index (BMI) was 29.8 kg/m^2^ (overweight). He had a round face with deep set eyes, prominent forehead, short fingers and small feet. The patient has had several full blood analysis, including platelets counts and volume, which resulted to be always within the normal range.

### Patient 3 (DECIPHER patient JFL255929, ClinVar SCV000328243)

He was the third child of young and unrelated parents, born at 38 WG with normal biometric values after a pregnancy complicated of polyhydramnios and minor cerebral ventriculomegaly. Cardiac and abdominal defects were excluded. He was referred for minor cognitive impairment with speech defect. He walked alone at 18 months. At 8 years and 9 months his height was −1 SD, the weight <−2 SD and OFC >+2 SD. He presented with macrodontia with teeth malposition, myopia and astigmatism, long palpebral fissures and pointed chin and a premature puberty (stage I according to Tanner scale). He had fingers pads and complex inner and middle ear abnormalities associated with conductive hearing deafness, improved by tympanoplasty. Platelets count was normal.

### Patient 4 (DECIPHER patient WLN265435, ClinVar SCV000328244)

He is the first child of unrelated parents, born from a intracytoplasmic sperm injection pregnancy because of polycystic ovarian disease and low sperm count. Pregnancy was complicated by intrauterin growth restriction (IUGR) and polyhydramnios. Delivery was induced at 38 WG and all body measurements were around or below −2 SD. He presented with developmental delay (especially expressive language delay), good receptive language and social ability, delayed achievement of motor milestones. He showed short stature, consistently below −2 SD, with normal OFC and delayed bone age. Ears were low set and posteriorly rotated. Among his facial dysmorphisms: broad nose with a bulbous tip, low nasal bridge and smooth philtrum, high and prominent forehead, frontal bossing, arched eyebrows, low nasal bridge and pointed chin were present. Single palmar crease right hand, proximally placed thumbs, rounded distal phalanx of thumbs and big toes were noted. The cardiac ultrasound (US) evaluation identified a small muscular and one membranous VSD with mildly dysplastic pulmonary valve. The computed tomography (CT) scan brain was normal. The patient has always had normal platelet counts and volume, at least until the age of 3 years and 2 months.

### Patient 5 (DECIPHER patient MCG251801, ClinVar SCV000328245)

This boy was born at term from uncomplicated pregnancy. In the first months of life, he developed gastro-esophageal reflux causing failure to thrive, which resolved by 2 years of age. He smiled at 8–9 weeks, sat independently at 8–9 months and walked independently at 18 months of age. He spoke his first recognizable words at 2 years 3 months of age. He came to medical attention at 2 years of age because of gastro-esophageal reflux, macrocephaly with mild plagiocephaly, developmental delay, right cryptorchidism, mild muscular hypotonia and some dysmorphic features (prominent forehead, round face, arched eyebrows, large and low set ears and broad nose, see Figure 1c). He suffered from recurrent blepharitis and bilateral sensorineural hearing loss. Blood tests were all normal.

### Patient 6 (ClinVar SCV000328246)

**Figure 2:**
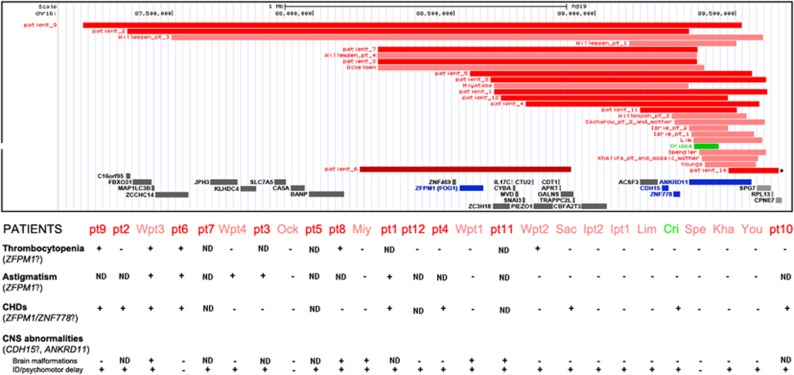
Correlation between the size of the microdeletions and the clinical features in the reported cases (11 sporadic and 2 familial, light red) and the 12 novel ones (dark red). The case with intragenic micoduplication is also reported in green. Genes in blue are those we considered mainly responsible for the clinical features in 16q24.3 microdeletions. Case 6 is the only one whose deletion does not include the *ANKRD11* gene.

This is the only patient for whom *ANKRD11* is not deleted (see Figure 2).

The boy was born by emergency cesarean section because of fetal distress after 37 WG. He suffered from perinatal asphyxia, cardiovascular and respiratory insufficiency, pulmonary hypertension and transient thrombocytopenia (platelets count – day 1: 29 × 10^9^/l, day 9: 18 × 10^9^/l; day 20: 51 × 10^9^/l, day 29: 128 × 10^9^/l, day 56: 329 × 10^9^/l and normal afterward). Bilateral adrenal hemorrhages resulted in adrenal insufficiency. A small muscular VSD and congenital hypothyroidism were detected. Magnetic resonance (MR) at age 1 year showed post asphyxia damage, in absence of cerebral structural anomalies. He wears glasses because of severe astigmatism. At the age of 6 years, he attends a regular school but needed extra support and had a short attention span. He showed mild dysmorphic features: a high forehead with frontal bossing, a deep nasal bridge and asymmetrically placed ears.

### Patient 7 (ClinVar SCV000328247)

The boy was born after an uneventful pregnancy and delivery. A diagnosis of pervasive developmental disorder not otherwise specified associated with developmental coordination disorder was made. His performal IQ was 67, his verbal IQ was 81. He presented with short stature (156.7 cm, −1 SD) and normal weight, OFC and BMI. He had mild facial dysmorphisms: round face with high and broad forehead, large incisors and a preauricular tag at the right side. He had proximally implanted thumbs. He wore glasses for astigmatism. He showed apraxia and a stiffened gait with retraction of Achilles tendons. Blood tests did not reveal any abnormal values. The boy followed special education till the age of 12, afterward he was able to switch to regular low-level education.

### Patient 8 (ClinVar SCV000328248)

This 29 year old man is the only child of unrelated parents. He was born at term by an emergency cesarean section because of breech presentation. His developmental milestones were slightly delayed: he smiled at 2 months, sat up at 9 months and walked unaided at 17 months. His speech was severely impaired as he started speaking at the age of 4. This problem was worsened by severe secretory otitis media, which required tympanostomy and grommets. At the age of 3, he underwent orchidopexy for undescended testes. At the age of 5, he was diagnosed with autism and attended a special needs schools. He suffered from epilepsy from the age of 15 years; at the onset seizures were generalized tonic–clonic with perioral cyanosis. Electroencephalography (EEG) showed left temporal abnormalities. From that time, he experienced about three to four episodes per year, sometimes occurring in clusters, despite antiepileptic therapy. Seizures disappeared by the age of 23 years. At the time of the first evaluation (25 years old), his physical examination showed clear dysmorphisms: forehead cleft with collapsed nasal bridge, bilateral ptosis (worse on the left), and a high, arched palate. He also presented with levoconvex scoliosis. Neurological examination showed anosmia, slow and hypometric saccades, impaired upgaze with frontalis overactivity, right exotropia and failure of convergence. He was markedly hypometric on finger–nose testing. His tendon reflexes were subdued. He also had a history of anxiety, angry outbursts and impulsivity. At the age of 20, his full scale IQ score was 74 (borderline intellectual functioning). Blood tests revealed a subclinical low platelet count (138 × 10^9^/l), low serum urea and creatinine. Cardiac and renal ultrasound examinations were normal. Video-telemetry EEG showed abnormal interictal activity with widespread slowing, on some occasions more evident over the right hemisphere. Frequent epileptiform abnormalities were present over the left temporal region and were rarely also seen on the right side. Cerebral MR showed a mega cisterna magna and biparietal parenchymal loss; the body of the left hippocampus demonstrated a slightly atypical morphology without a definite malformation or signal abnormality.

### Patient 9 (ClinVar SCV000328249)

This is the third child of healthy, unrelated parents. He was born at a gestational age of 38 WG by C-section because of IUGR, VSD and fetal distress. At birth, all his measurements were below −2 SD. At the age of 1 month, low platelets count was observed (40 × 10^9^/l). Clinical examination at age of 3 years and 3 months revealed: weight 10.7 kg (< −2 SD), height 85.3 cm (< −2 SD) and OFC 49.7 cm (normal). His psychomotor development was mildly delayed. He had mild bilateral eyelid ptosis and one café-au-lait spot on the left leg. His cerebral MR imaging revealed a delayed myelination.

### Patient 10 (ClinVar SCV000328250)

She was referred at 7 months and then at 22 months for the association between global developmental delay and congenital heart defects (CHDs). OFC was below −2 SD both at 7 months (39.9 cm) and at 22 months (42.8 cm). She did not showed any dysmorphic facial features. Cardiac US assessment identified: hypoplasia of left ventricle, aortic annulus, aortic arch, cleft mitral valve with a combination of mitral stenosis and insufficiency. Pulmonary hypertension was resolved after mitral valve surgery at 9 months. Normal platelets count was observed. She manifested a possible seizure episode at 4 years of age, consisting in tonic extension with fixed gaze followed by lethargy. The EEG showed a diffuse, very slow background activity, slow occipital rhythm, lateralization to the left occipital-posterior lobe temporal activity. A CT scan of the brain at 4 years of age showed a possible underdevelopment of the inferior cerebellar vermis. At the same age, her general condition worsened, the patient went into shock and died some months after.

### Patient 11 (DECIPHER patient PAR259954, ClinVar SCV000328251)

She is the fifth child of healthy parents. Parents are first-degree cousins and all sisters and brothers are healthy. The pregnancy and the delivery were uneventful. She had motor skill delay (sitting alone at 2 and a half years; walking alone at 4 years and 6 months). She had absent language and autistic symptoms: poor eye contact and interactions with others, stereotypies with hand flapping and bruxism, self-injurious behavior and sleep difficulties. She presented with facial features characterized by macrodontia, synophris, hypertelorism, large bulbous nose with anteverted nares and small extremities (Figures 1d and e). Blood tests showed normal values of platelets count. She had chronic rhinitis. She had drug-resistant generalized seizures since 1 year of age, and cerebral MR revealed bilateral atrophy of the cerebral white matter.

### Patient 12 (ClinVar SCV000328252)

The patient was born at 40 WG after an uncomplicated pregnancy; family history was unremarkable. Her motor development was normal but cognitive development was slow. She was evaluated at the age of 32 years because of psychiatric problems, showing an IQ of 65. At the age of 45 years, she underwent surgical correction of hip dysplasia, responsible of coxarthrosis. At the age of 48 years, she was re-evaluated at the department of clinical genetics because of intellectual disability and automutilation problems. She showed different skin lesions, such as alopecia areata on the head, psoriasis of the extremities and at least one episode of erysipelas in the previous years. She wore hearing aids and glasses because of the severe myopia. Recently she had had an operation of her left eye because of a mild facialis paresis.

At that time her height was 163.5 cm (−1 SD), weight 65 kg (+1 SD) and OFC 56 cm (+1 SD). She was remarkably shy and her face was round with a low frontal hair line, broad nose at the basis, long smooth philtrum, cupid shape of the mouth with downturned corners of the mouth (Figure 1f), and cutaneous syndactyly of toes II and III and of fingers II/III, III/IV and IV/V. Investigations (blood tests, EEG, CT scan skull) were all normal.

## Methods

Array-CGH was performed with different platforms (see Supplementary Table S2). Only in one case BAC array was used; in the latter case no further studies have been done. All data are reported according to February 2009 assembly, GRCh37/hg19. Data confirmation were performed by Q-PCR or FISH analysis (Supplementary Table S2) with specific primers or probes (data not shown) and extended to parents.

## Results

### Molecular and clinical results

Supplementary Tables S1 and S2 recapitulate the deletion size and the clinical features of the subjects with 16q24 microdeletions already reported and of the 12 patients presented in this report, respectively. A graphical overview of the same subjects is provided in Figure 2; the deletion size for case 10 is unclear because an old BAC array showed a single clone deletion for probe RP11-104N10 and no further studies could be done (see * in Figure 2). The 16q24.2q24.3 deletions were *de novo* in all the 11 cases we could analyze.

A comprehensive overview of main clinical features of patients with either *ANKRD11* variants, *ANKRD11* deletion/duplication or 16q24.3 microdeletion is presented in Table 1, clearly showing that only individuals with 16q24 microdeletion syndrome may manifest astigmatism or thrombocytopenia. Although the small number of cases prevent statistical significance, both the frequency of CHDs and CNS malformation show an higher incidence in patients with 16q24 microdeletion syndrome with respect to those with KBG syndrome.

## Discussion

Since 2011, KBG syndrome has been attributed to *ANKRD11* haploinsufficiency.^2^ This gene encodes an ankryin repeat domain-containing protein, which acts as a transcriptional coregulator and has been shown to be crucial for neural development.^18^ To the best of our knowledge, 23 *ANKRD11* variants leading to KBG syndrome have been described^2, 3, 6, 7, 10^ and other 2 *ANKRD11* variants were reported in patients with a more complex genotype.^8, 9^ Most of these variants are private, except for NM001256182.1c.1903_1907del highlighted in three unrelated families,^3, 6^ and are predicted to result in LoF of the protein. Many *ANKRD11* variants generate premature stop codons, leading to the formation of a truncated protein. Recent insights about the possible pathological mechanism underlying KBG syndrome have shown that such truncated protein would accumulate inside the cell, by escaping both mRNA decay process and proteasome-mediated degradation.^6^ As ANKRD11 produces homodimers through N-terminus interactions, the accumulation of the aberrant form of the protein may suggest a dominant-negative mechanism, as already proved in the murine model for KBG syndrome.^6^

Recently, a familial intragenic duplication of *ANKRD11*, predicted to result in a truncated protein, has been associated with KBG syndrome, reinforcing the evidence of a dominant-negative mechanism.^5^ Besides intragenic variants, KBG syndrome can also be caused by microdeletions encompassing part or the entire *ANKRD11* gene. Up to now six individuals with such a deletion have been identified.^4, 11, 12, 13, 14^ Three of these deletions include the *SPG7* gene as well,^4, 13, 14^ situated distally to *ANKRD11*; homozygous *SPG7* variants are associated with spastic paraplegia 7 (MIM #607259). Although a possibly dominant effect has been suggested recently for some single heterozygous variants, reports that deletions of this gene are pathogenic only when homozygously present, make it unlikely that there is any effect of *SPG7* haploinsufficiency on the phenotypes of the cases reported here.^19^

A phenotype overlapping the KBG syndrome has been described for 16q24.3 microdeletions. After the first report of four cases by Willemsen *et al*,^16^ other four patients have been reported involving, at least in part, the *ANKRD11* gene and extending more proximally up to 2 Mb, encompassing among others, the *ZFPM1*, *CDH15* and *ZNF778* genes.^3, 15, 17^ Recently, it has been questioned whether haploinsufficiency for flanking genes may contribute to a more complex or a more severe phenotype.^3^

From the detailed genotype–phenotype investigation of our cohort of 12 new cases with 16q24.3 microdeletions, we propose a tentative correlation between some of the deleted genes and few specific traits, identifying those distinctive clinical features possibly distinguishing 16q24.3 deletion from KBG syndrome.

### Thrombocytopenia

The *ZFPM1* (*FOG1*) gene, mapping about 730 kb upstream to *ANKRD11*, is deleted in our patients 2, 3, 5, 6, 7 and 9 (Figure 2). This gene encodes a nine-zinc-finger transcriptional regulator required for a proper differentiation and maturation of both megakaryocytes and erythroid precursors,^20^ acting in concert with the hematopoietic master regulator *GATA1*.^21^ The murine model knockout either for *ZFPM1* or *GATA1* shows a similar lethal phenotype, consisting of thrombocytopenia and severe anemia.^22^ Not surprisingly, three of the deleted patients (our cases 6 and 9, and patient 3 in Willemsen *et al*)^16^ and the two with a breakpoint downstream *ZFPM1* (our case 8 and patient 2 in Willemsen *et al*)^16^ at 29 and 670 kb, respectively, present with subclinical thrombocytopenia. For these latter two patients, a position effect, altering *ZFPM1* expression, appears very likely. Accordingly, the region downstream *ZFPM1* gene results to be quite conserved in mammals (Supplementary Figure S1) and contains ZFPM1:miR-106/302, a conserved mammalian microRNA regulatory target site. Unfortunately, having gathered the cases from different medical centers, biological samples to assess this point through specific expression studies were not available.

Although most of the *ZFPM1* deleted patients did not show bleeding disorders, the role of *ZFPM1* in hematopoiesis is reinforced by the demonstration that knockout mice are severely affected by thrombocytopenia.^23^ On the other hand, many variants in genes related to thrombocytopenia show variable expression.^24^ Moreover, *ZFPM1* and *GATA1* interact both functionally^25^ and physically,^26^ and disruption of the normal interaction of *ZFPM1* and *GATA1* has been linked to a range of inherited blood disorders.^27^

It is interesting that a paralog of *ANKRD11*, namely *ANKRD26*, is linked to thrombocytopenia inherited in a dominant manner and that *ANKDR11* itself is expressed in bone marrow at high level. However, the finding that none of the 38 KBG subjects, either mutated or deleted for *ANKDR11*, showed thrombocytopenia makes unlikely its involvement in this disorder (Table 1).

### Astigmatism

*ZFPM1* (*FOG1*) is also crucial for a correct eye development through the interaction with other *GATA* factors and its haploinsufficiency seems to be associated with severe astigmatism, as reported in our cases 1, 3, 6 and 7, and patients 3 and 4 in Willemsen *et al*^16^ (Supplementary Tables S1 and S2, Figure 2). In particular, *GATA3* seems to be strongly involved in correct lens development in the murine model, with a specific expression in lens fibers. Moreover, *Gata3* inactivation leads to a reduced differentiation and abnormal apoptosis of the posterior lens fiber cells.^28^ On the other hand, overexpression of the fly homolog for FOG genes, Ush, results in aberrant eye differentiation, probably by inhibition of the fly homolog for GATA4, Pnr.^29^ These two lines of evidences suggest a dosage-sensitive mechanism underlying FOG-GATA-dependent eye development. In this sense, the presence of severe astigmatism in patients 3 and 4 in Willemsen *et al*^16^ and in our cases 3 and 6 may be explained by an impairment of *ZFPM1* function. Similarly, the lens defect found in our patient 1, whose deletion breakpoint falls at 41 kb downstream to the 3′ of *ZFPM1*, may be explained in term of disruption of regulatory elements of the gene, as already hypothesized above for thrombocytopenia.

### Congenital heart defects

FOG/GATA interaction has been demonstrated to be essential also for heart embryogenesis. In zebrafish, ZFPM1 mutants show aberrant cardiac looping and pericardial edema secondary to hypoplastic ventricular wall looping.^30^ In *Drosophila,* Ush overexpression causes inhibition of cardiac cell proliferation, producing gaps in the heart tube.^29^ Murine model knockouts for Gata4 die at 9.5 days post coitum because of cardiac malformations.^31^ In humans, *GATA4* and *GATA6* (OMIM *600576 and *601656) haploinsufficiency are known to be associated with CHDs, and in particular with VSDs. Taken together, all of these reports suggest a possible role of *ZFPM1* deletion in causing CHDs. In fact, we report a higher incidence of CHDs for 16q24.3 microdeletion patients (patient 3 in Willemsen *et al*,^16^ and our cases 1, 2, 4, 6 and 9, about total 40% of cases) in comparison with KBG subjects (10%).

As discussed, *ZFPM1* haploinsufficiency causes severe perturbations during developmental stages, affecting in particular the hematopoietic system, the eye and the heart. Crawford *et al*^32^ demonstrated that in mice *Pbp* null mutations, coding for the peroxisome proliferator activator receptor-binding protein, produce very similar defects in embryogenesis, consisting of ventricular myocardium disruption, eye aberrations and impaired hematopoiesis. The authors demonstrated that PBP interacts with all GATA factors and that the lethal murine phenotype is due to the dysfunction of factors within the GATA family. The evidence of a common and reproducible phenotype caused by a functional impairment in GATA elements, because of the haploinsufficiency of one of their interactors, gives further support to the possible contribution of *ZFPM1* in the genesis of the 16q24.3 microdeletion phenotype.

Another gene deleted in 16q24.3 microdeleted patients with CHDs is *ZNF778,* which belongs to the zinc-finger protein family and is widely expressed both in the brain and in the heart. For its function of transcriptional regulator, it is predicted to interact with GATA4 that, as discussed above, has been demonstrated to be fundamental in heart development (Supplementary Figure S2). In this sense, haploinsufficiency for *ZNF778* may also contribute to the higher frequency of CHDs, essentially VSDs, present in the 16q24 microdeletion syndrome compared with KBG syndrome. For example, Sacharow *et al*^15^ described a patient with a VSD and supravalvular pulmonary stenosis, for whom a deletion involving only *ANKRD11* and *ZNF778* was identified. On the other hand, four out of six of our cases presenting with CHDs are deleted for *ZNF778* (cases 1, 2, 4 and 9).

### The neurological phenotype

Patients with KBG syndrome because of a LoF variants in *ANKRD11* gene may show: mild-to-moderate intellectual disability, epilepsy or EEG anomalies, brain malformations, behavioral and autistic spectrum disorders. On the contrary, KBG patients because of encompassing only *ANKRD11* have been reported, on average, to show milder neurological involvement with developmental delay mostly limited to language impairment, with borderline cognitive skills, lower frequencies of epilepsy, autism spectrum disorders and CNS structural aberrations (not all underwent neuroimaging studies). The milder phenotype in patients with *ANKRD11* haploinsufficiency compared with those with an intragenic variant may be explained by the dominant-negative effect of the latter as suggested by Walz *et al.*^6^

The putative role of the *CDH15* gene for the neurological burden is particularly relevant in term of structural brain abnormalities, as all five patients presenting with such cerebral findings carry a 16q24.3 microdeletion including the *CDH15* gene (Figure 2 and Table 1). The *CDH15* gene encodes cadherin 15, a calcium-dependent cell adhesion molecule belonging to the cadherin family. Cadherins are expressed in the entire human brain and their dysregulation so far has been associated with many neuropsychiatric disorders, including intellectual disability, schizophrenia and epilepsy.^33^ Regarding specifically *CDH15*, Bhalla *et al*^34^ identified four heterozygous non-synonymous variants in unrelated female patients with mild-to severe intellectual disability. Functional studies demonstrated reduced cell adhesion in murine cells expressing mutated *CDH15*. However, in one case the variant had been inherited from a healthy parent, suggesting incomplete penetrance. The observation of a worse neurological phenotype for larger deletions suggests a possible role for *CDH15* in regulating proper CNS embryogenesis.

Remarkably, some patients with a *CDH15* deletion share specific clinical traits with CHARGE syndrome, associated with heterozygous *CDH7* variants. In particular, cases 8, 9 and the case described by Miyatake *et al*^17^ presented with ptosis, whereas case 8 presented anosmia as well.

### Teeth anomalies

Impaired dental development is considered as the most specific clinical sign for KBG syndrome and thus it has been listed among the major diagnostic criteria. Different anomalies have been identified: macrodontia of the permanent upper central incisors (as defined by Moyers^35^), hypodontia, supernumerary mammelons, cleft teeth and enamel hypoplasia or pits^1^ have all been documented. Such alterations have been reported for almost all individuals with KBG syndrome independently of the underlying molecular defect, namely *ANKRD11* variant or deletion, although for two patients with an *ANKRD11* deletion no permanent teeth anomalies were reported.^12^

The dental dysmorphisms reported in patients with 16q24.3 microdeletion are very similar to those described for *ANKRD11* haploinsufficiency, albeit occurring with a lower frequency (Table 1). It appears difficult to gather all this data in a comprehensive genotype–phenotype framework and such variability may be due to the effect of other modifier genes in the microdeletion.

### Miscellaneous

Patient 9 carries the most proximal deletion of our cohort and his phenotype is characterized also by renal malformation. Handrigan *et al*^36^ reported an augmented incidence of renal malformations in patients with deletion of the region 16q24.1q24.2. The area of minimal overlap includes the genes *FBXO31*, *MAP1LC3B* and *ZCCHC14* and the deletion of our patient 9 extends to the distal end of the *ZCCHC14* gene. It is then arguable that his kidney defect may be related to the involvement of this region, possibly critical for renal development. Crippa *et al*^5^ described two related KBG patients with an intragenic *ANKRD11* duplication. One of them had third-degree vesicoureteral reflux and the other had left ureterocele and duplex pelvicalyceal district. Renal abnormalities were not previously reported for the exclusive involvement for *ANKRD11*, but the authors could not exclude a random association.

In our cohort, patient 6 shows a proximal deletion not involving the *ANKRD11* gene and including only *ZFPM1*. His phenotype includes: perinatal asphyxia, normal cognitive level, speech delay, no structural anomalies in brain development, normal height and OFC, absent facial dysmorphisms, neonatal transient thrombocytopenia, VSD and severe astigmatism. This clinical presentation is quite different from those because of larger deletions and supports the role of *CDH15*, *ZNF778* and *ANKRD11* in the 16q24.2 microdeletion syndrome and the importance of *ZFPM1* for cardiac, hematopoietic and eye maturation.

In conclusion, the comparison between the clinical features of 26 patients with 16q24.2q24.3 deletion of different sizes and 32 *ANKDR11* mutated patients, show that astigmatism and thrombocytopenia are present exclusively in patients with the deletion involving *ZFPM1* or with breakpoints spanning up to it, as it clearly appears from Table 1. Moreover, *CDH15* haploinsufficiency may contribute to a more severe neurological phenotype, with particular regard to brain malformations, whereas *ZFPM1* and *ZNF778* haploinsufficiency may results in an increased risk for CHDs. *ZFPM1* seems also to be crucial for correct bone marrow function as deletion for this gene seems to result in (asymptomatic) thrombocytopenia with reduced penetrance.

All these features should be taken into account to distinguish between KBG and 16q24 microdeletion syndromes.

## Figures and Tables

**Table 1:** Summary of the main clinical features in unrelated subjects with either isolated *ANKRD11* alterations or 16q24.3 microdeletion^a^

*Clinical features*^b^	*ANKRD11 mutation*^c^	*ANKRD11 deletion*^d^	*ANKRD11 intragenic duplication*	*16q24.3 Microdeletion syndrome*		
				*Previous report*	*This report*^e^	*Total*
Gender	22 M 10 F	5 M 1 F	1 M 1 F	7 M 1 F	7 M 3 F	14 M 4 F
Characteristic facial anomalies	32/32 (100)	6/6 (100)	2/2 (100)	5/8 (62.5)	3/10 (30)	8/18 (44.4)
Macrodontia of upper central incisors	29/32 (90.6)	3/6 (50)	2/2 (100)	4/8 (50)	5/10 (50)	9/18 (50)
Postnatal short stature with height <3rd centile	20/32 (62.5)	5/6 (83.3)	2/2 (100)	2/8 (25)	4/10 (40)	6/18 (33.3)
Mild-to-moderate developmental delay	11/32 (34.4)	5/6 (83.3)	0/2 (0)	7/8 (87.5)	8/10 (80)	15/18 (83.3)
Mild-to-moderate intellectual disability	26/32 (81.3)	1/6 (16.7)	2/2 (100)	3/8 (37.5)	7/10 (70)	10/18 (55.6)
Seizures	7/32 (22)	1/6 (16.7)	0/2 (0)	3/8 (37.5)	2/10 (20)	5/18 (27.8)
**CNS**^f^ **malformation**	**1/32 (3.1)**	**0/6 (0)**	**0/2 (0)**	**3/8 (37.5)**	**2/10 (20)**	**5/18 (27.8)**
Hand anomalies	29/32 (90.6)	4/6 (66.7)	2/2 (100)	3/8 (37.5)	2/10 (20)	5/18 (27.8)
Costovertebral anomalies	17/32 (53.1)	0/6 (0)	0/2 (0)	0/8 (37.5)	0/10 (0)	0/18 (0)
Significantly delayed bone age	10/32 (31.3)	2/6 (33.3)	0/2 (0)	2/8 (25)	3/10 (30)	5/18 (27.8)
Cryptorchidism	11/22 (50)	0/5 (0)	0/1 (0)	2/7 (28.6)	3/7 (42.9)	5/14 (35.7)
**Thrombocytopenia**	**0/32 (0)**	**0/6 (0)**	**0/2 (0)**	**2/8 (25)**	**2/10 (20)**	**4/18 (22.2)**
**Severe astigmatism**	**0/32 (0)**	**0/6 (0)**	**0/2 (0)**	**2/8 (25)**	**3/10 (30)**	**5/18 (27.8)**
**CHDs**^g^	**3/32 (9.4)**	**0/6 (0)**	**2/2 (100)**	**2/8 (25)**	**4/10 (40)**	**6/18 (33.3)**
First-degree relative with KBG	11/32 (34.4)	1/6 (16.7)	2/2 (100)	2/8 (25)	0/11 (0)	2/18 (11.1)

Bold values indicates the clinical features that distinguish 16q24.3 microdeletion syndrome from KBG syndrome.

a Patient 6 was excluded because *ANKRD11* is not involved in the deletion region; patient 10 was excluded as well, being deletion was distal to *ANKRD11.*

b According to Skjei *et al*, revisited.

c References: ^2, 3, 6, 7, 10^.

d References: ^4, 11, 12, 13, 14^.

e Only six out of seven patients were considered as one presented the deletion in mosaic.

f Central nervous system.

g Congenital heart defects.
