# Supplementary material for: Haploinsufficiency for ANKRD11-flanking genes makes the difference between KBG and 16q24.3 microdeletion syndromes: 12 new cases
Source: Eur J Hum Genet. 2017 Apr 19;25(6):694–701. doi: 10.1038/ejhg.2017.49 (PMC5533198; doi:10.1038/ejhg.2017.49)
Supplement: Supplementary Table S1 [file ejhg201749x1.docx]

Supplementary Table 1 - Detailed clinical features of microdeletion patients: 11 sporadic and 2 familial already reported.

|  | **Marjolein H Willemsen et al., 2010** | | | | **Youngs et al., 2011** | **Isrie et al., 2012** | | **Miyatake et al., 2013** | **Sacharow et al., 2012** | | **Khalifa et al., 2013** | | **Lim et al., 2014** | **Spengler et al., 2013** | **Ockeloen et al., 2015** |
| --- | --- | --- | --- | --- | --- | --- | --- | --- | --- | --- | --- | --- | --- | --- | --- |
|  | Patient 1 | Patient 2 | Patient 3 | Patient 4 |  | Patient 1 | Patient 2 |  | Patient 2 |  |  |  |  |  | patient 13 |
| **16q24 deletion size (the minimum extent of the deletion is reported)** | 378 Kb | 265 Kb | 2.07 Mb | 1.1 Mb | 180 Kb | 221 Kb | 137 kb | 690 kb | 320 kb | 320 Kb | 197Kb | 197Kb in mosaic |  | 348 kb | 1.16 Mb |
| **Mb position (hg19)** | 89.12–89.50 | 89.27–89.53 | 87.05–89.6 | 88.23–89.36 | 89.39-89.57 | 89.34-89.56 | 89.33-89.47 | 88.64-89.33 | 89.28-89.60 | 89.28-89.60 | 89.38-89.58 | 89.38-89.58 | 89.35-89.59 | 89.37–89.60 | 88.23-89.39 |
| **Sex** | M | M | M | M | M | F | M | M | M | F | M | F | M | M |  |
| ***De novo/***  **inherited** | *de novo* | *de novo* | *de novo* | *de novo* | *de novo*? (father not available) | *de novo* | *de novo* | *de novo* | inherited from the mother | mother of patient | inherited from the mother (the mother is mosaic) | mother of the patient | *de novo* | *de novo* | *de novo* |
| **Age at examination** | 22 years | 3 years 3 months | 6 years 3 months | 8 years 10 months | 17 years | 5 years | 19 years | 4 years | 5 years |  | 2 years 6 months |  | 6 years 6 months | 4 years |  |
| **Height** | 3–10th percentile | 10–25th percentile | 5–12th percentile | 10–25th percentile | <3rd percentile | nd | 165 cm (<3rd percentile) | 100.6 cm (25th-50th percentile) | 113 cm (50th) | short stature | 10th percentile | short stature | short stature less than 3rd percentile | <3rd percentile | short stature |
| **Head circumference** | 90th percentile | 50th percentile | 10th percentile | 50–75th percentile | <3rd percentile | nd | 57.9 cm (50th-75th percentile) | 51.5 cm (75th percentile) | - | not reported | 75th percentile | 55.5 cm | not reported | 90th-97th percentile | unknown |
| **Facial features** |  |  |  |  |  | abnormal facial shape |  | KBG-like |  |  |  |  | KBG-like |  | + |
| **high forehead** | + | - | + | + | + | - | + | - | - | + | - | - | - | - |  |
| **prominent forehead** | - | - | - | - | + | plagiocephaly | - | - | - | - | - | - | + | + |  |
| **frontal bossing** | - | + | + | - | - | - | - | - | - | - | - | - | - | - |  |
| **bitemporal narrowing** | + | - | - | - | - | - | - | - | - | - | - | - | - | - |  |
| **long ovale face** | + | - | - | - | - | - | - | - | - | - | - | - | - | - |  |
| **round face** | - | - | - | - | - |  | - | - | + | - (triangular face) | + | + | - (triangular face) | - |  |
| **long palpebral fissures** | + | + | - | - | - | eyes far from each other | - | eyes far from each other | + | + | + | - | - | - |  |
| **slanted palpebral fissures** |  |  |  |  | midly upslanted |  |  |  |  |  |  | - | - | - |  |
| **arched eyebrows** | - | - | + | - |  | - | - | wide eyebrows with synophrys | eyebrows laterally flared | - | + | - | - | - |  |
| **deep set eyes** | - | - | - | - |  | - | + | - | - | - | - | - | - | - |  |
| **large ears** | + | + | + | + | + | protruding ears | protruding ears | - | - | - | - | - | - | + |  |
| **low set ears** | - | - | - | - |  | - | - | - | - | - | - | - | - | + |  |
| **broad nose** | - | - | - | - |  | large nose | + | - (nteverted nostrils) | - | - | - | - | - | - |  |
| **low nasal bridge** | - | - | - | - |  | - | - | - | - | - | - | - | - | - |  |
| **high nasal bridge** | - | - | - | - |  | - | - | - | - | - | - | - | - | - |  |
| **smooth philtrum** | + | + | + | - |  | - | - | broad philtrum | - | - | + | - | - | - |  |
| **broad mouth** | + | + | + | - |  | - | + | - | + | - | - | - | + | - |  |
| **pointed chin** | + | + | + | - |  | - | + | - | - | + | - | - | - | + |  |
| **micrognathia** | + | + | - | - |  | - | - | - | - | - | - | - | - | - |  |
| **high palate** | + | - | + | - |  | - | - | - | - | - | + | + | - | - |  |
| **Macrodontia** | - | - | - | + | + | - | - | + | - | + | + | + | + | - | + |
| **Addiotional dental abnormalities** | large front teeth |  |  | fusion of teeth | dental crowding, bilateral fusion central and lateral upper incisors |  |  | wide lower incisors, oligodontia, central clefts | large central incisors In primary dentition |  |  | malposition and extra teeth |  |  | Large upper lateral incisors Unusually shaped teeth |
| **Cognitive impairment** | moderate ID | normal nonverbal IQ with moderate-severe speech delay | moderate ID | borderline-normal verbal IQ, mildly impaired nonverbal IQ | mild to moderate ID | delayed speech and language development, growth retardation | borderline ID (IQ of 77, WISC-R, evaluated at the age of 9 years) | developmental delay, moderate ID (Developmental Quotient 40), speech delay | ID, speech delay | learning disability | ID, greatest areas of expressive language | learning difficulty in her early school years | developmental delays in speech and motor functions, IQ 70 | normal | developmental delay |
| **Autism spectrum disorder** | borderline | + | + | + | + | - | - | - | - | - | - | - | - | - | - |
| **Attention deficit disorder** | - | - | - | - | - | '- | + | - | + | - | - | - | - | - | - |
| **Seizures** | absences and generalized | - | partial complex | - | - | - | + | asymptomatic multifocal epileptic discharges | - | - | febrile seizures, normal EEG | - | - | - | Atypical seizures at 3 months with EEG abnormalities |
| **Structural brain malformation** | corpus callosum hypoplasia, colpocephaly | - | corpus callosum hypoplasia, dilated ventricles, optic nerve hypoplasia | NT | - | - | - | hypoplasia of the cerebellar vermis; decreased white matter in both sides of cerebral hemispheres | - | - | - | - | - | - | mildly delayed myelination |
| **Neuronal migration disorder** | heterotopias | normal unenhanced CT cerebrum | perventricular heterotopias (unilateral) | NT | - | - | - | - | - | - | - | - | - | - | - |
| **EEG abnormalities** | - | - | - | - | - | - | - | - | - | - | - | - | - | - | - |
| **Ocular problems** | strabismus | - | high myopia, astigmatism, horizontal nystagmus | severe bilateral astigmatism | - | - | - | ptosis | - | - | - | - | - | - | - |
| **Hearing loss** | - | - | mixed sensorineural and conductive hearing loss | - |  |  | slightly impaired at one side | - | mild conductive hearing loss |  | chronic otitis media; several hearing studies but not conclusive |  |  | - | - |
| **Congenital heart defect** | - | - | VSD, PFO and cleft mitral valve with severe mitral regurgitation | NT | - | thick mitral valve | - | - | VSD, supravalvular pulmonic stenosis | - | PFO | - | - | - | - |
| **Skeletal anomalies** | kyphoscoliosis | - | - | - | - | - | - | - | - | - | - | - | narrowing and elongation of both iliac wings and body, small femoral heads, small distal tibial epiphyses and tibiotalar slanting | - | not reported |
| **Hand/foot anomalies** | - | - | - | - | - | clinodactyly, syndactyly | short metacarpals of the fourth and fifth fingers of his left hand | brachydactyly of the fifth fingers; | mild 5th finger clinodactyly | small hands with short fingers and mild 5th finger clinodactyly, brachydactyly and partial syndactyly between the 2nd and 3rd toes | small hands with brachydactyly; partial sindactyly 2nd-3rd toes | postaxial polydactyly on the left hand then removed |  | - | - |
| **Genital anomalies** | - | - | unilateral cryptorchidism | - | - | - | - | - | cryporchidism | - | hypospadias with cordee and penile-scrotal fusion | - | - | - | NA |
| **Hematologic disorder** | - | neonatal thrombopenia (resolved) | thrombopenia, macrocytosis (resolved) | - | - | - | - | - | - | - | - | - | - | - | - |
| **Delayed bone age** | - | - | - | - | unknown | - | - | + | - | - | + | - | + | - | not reported |
| **Others** | - | - | - | - | - | flat thorax, torticollis, motor deterioration, short-broad neck | mild synophrys, short columella, | mild truncal ataxia | - | - | umbilical hernia, intestinal malrotation, nasal tone of voice | - | - | - | - |
