# Supplementary material for: Haploinsufficiency for ANKRD11-flanking genes makes the difference between KBG and 16q24.3 microdeletion syndromes: 12 new cases
Source: Eur J Hum Genet. 2017 Apr 19;25(6):694–701. doi: 10.1038/ejhg.2017.49 (PMC5533198; doi:10.1038/ejhg.2017.49)
Supplement: Supplementary Table S2 [file ejhg201749x2.docx]

Supplementary Table 2 - Detailed clinical features of microdeletion patients: the 12 novel ones.

|  | **Our paper** | | | | | | | | | | | |
| --- | --- | --- | --- | --- | --- | --- | --- | --- | --- | --- | --- | --- |
|  | Case 1  (ClinVar SCV000328241) | Case 2 (DECIPHER patient SMB25532 -  ClinVar SCV000328242) | Case 3 (DECIPHER patient JFL255929 - ClinVar SCV000328243) | Case 4 (DECIPHER patient WLN265435 - ClinVar SCV000328244) | Case 5 (DECIPHER patient MCG251801 - ClinVar SCV000328245) | Case 6  (ClinVar SCV000328246) | Case 7  (ClinVar SCV000328247) | Case 8  (ClinVar SCV000328248) | Case 9  (ClinVar SCV000328249) | case 10 (DECIPHER patient BAY249412 - ClinVar SCV000328250) | case 11 (DECIPHER patient PAR259954 - ClinVar SCV000328251) | case 12  (ClinVar SCV000328252) |
| **16q24 deletion size**  **(the minimum extent of the deletion is reported)** | 970 Kb | 1.99 Mb | 1.1 Mb | 829 Kb | 1 Mb | 748 kb | 1133 kb | 977 kb | 2.34 Mb | Unclear deletion size | 343 kb | 806 kb |
| **Position** (hg19, NC_000023.10) | chr16:g.( 88635000_88643461)_(89611494_89628950)del | chr16:g(?_ 87340135)_(89335428_?)del | chr16:g(?_88230961)_(89363602_?)del | chr16:g(?_ 88755312)_(89584412_?)del | chr16:g(?_ 88556191)_(89557911_?)del | chr16:g(?_ 88165980)_(88914268_?)del | chr16:g(?_ 88230760)_(89363742_?)del | chr16:g.(?_88630607)_(89607742_?)del | chr16:g. (?_87183661)_(89520803_?)del | chr16:g. (?_89475451)_(89652148_?)del | chr16:g. (?_89161684)_(89505106_?)del | chr16:g. (?_88666177)_(89472627_?)del |
| **Array platform** | Aglilent 180K (AMADID 022060) |  | Aglilent 60K (AMADID 031746) |  | BlueGnome 0.5Mb Cytochip (v3.1) | Agilent 180K custom (Oxford design, AMADID 023363) | Agilent 105K custom (Oxford design, AMADID 019015) | Nimblegen 135K WG CGH 1.3 | 180K Cytosure ISCA v2 array (OGT) | old BAC array showed a single clone deletion RP11-104N10, no further studies done. |  | Agilent 180K (AMADID 023363) |
| **Sex** | M | M | M | F | M | M | M | M | M | F | F | F |
| ***de novo/***  **inherited** | *de novo* | *de novo* | *de novo* | *de novo* | *de novo* | *de novo* | *de novo* | *de novo* | *de novo* | *de novo* | *de novo* | unknown |
| ***De novo* confirmation by** | FISH |  | QPCR | FISH | FISH | FISH |  |  | FISH |  |  | - |
| **Birth** | W 3050 gr  L 50 cm  OFC 34,5 cm  Apgar 9/10 | W ~2000 gr (<-2 SD) | W 3430 gr  L 50 cm  OFC 35 cm | W 2200 gr  (<-2 SD)  L <-2 SD  OFC 32.3 cm  (<-2 SD)  Apgar 9/10 | W 4100 gr | ND | W 3850 gr | Normal | W 1940 gr  L 42 cm  OFC 32 cm  Apgar 5/8 | ND | ND | ND |
| **Age at examination** | 8 years 6 months | 26 years | 8 years 9 months | 3 years 2 months | 2 years | 14 months | 13 years | 29 years | 3 years 3 months | 7 years 2 months | 14 years | 48 years |
| **Height** | < -2 SD | < -1 SD | -1 SD | < -2 SD | < -1 SD | 74.6 cm (normal) | 156.7 cm  -1 SD | normal | 85.3 cm  (< -2 SD) | unknown | 145 cm  (< -2 SD) | 163.5 cm (normal) |
| **Head circumference** | normal | > +1 SD | > +2 SD | normal | > +1 SD | 45.5 cm (normal) | 55 cm (normal) | normal | 49.7 cm (normal) | 7th month: 39.9cm  (<-2 SD)  22th month: 42.8 cm  (<-2 SD) | 53 cm  < -1 SD | 56 cm (normal) |
| **Facial features** |  |  |  |  |  |  | KBG-like |  |  | patients showed no dysmoprhic facial features, this patient deceased |  |  |
| **high forehead** | - | - | - | + | - | + | + | - | - | - | - | - |
| **prominent forehead** | + | + | - | + | + | - | + | - (forehead cleft) | - | - | - | - |
| **frontal bossing** | - | - | - | + | - | + | + | - | - | - | - | - |
| **bitemporal narrowing** | - | - | - | - | - | - | - | - | - | - | - | - |
| **long ovale face** | - | - | - | - | - | - | - | - | - | - | - | - |
| **round face** | - | + | - | - | + | + | + | - | - | - | - | + |
| **long palpebral fissures** | - | - | + | - | - | - | - | - | - | - | + | - |
| **slanted palpebral fissures** |  |  |  |  |  |  |  |  |  |  |  |  |
| **arched eyebrows** | - | - | - | + | + | - | - | - | - | - | - | - |
| **deep set eyes** | - | + | - | - | - | - | - | - | - | - | - | - |
| **large ears** | - | - | - | - | + | - | - | - | - | - | - | - |
| **low set ears** | - | - | - | + | + | - | -  (right tag) | - | - | - | - | - |
| **broad nose** | + | - | - | + | + | - | - | - | - | - | + | + |
| **low nasal bridge** | - | - | - | + | - | + | + | - (collapsed nasal bridge) | - | - | - | - |
| **high nasal bridge** | + | - | - | - | - | - | - | - | - | - | + | - |
| **smooth philtrum** | - | - | - | + | - | - | - | - | - (broad philtrum) | - | - | + |
| **broad mouth** | - | - | - | - | - | - | - | - | - | - | + | - |
| **pointed chin** | - | - | + | + | - | - | + | - | - | - | - | - |
| **micrognathia** | - | - | - | - | - | - | - | - | - | - | - | - |
| **high palate** | - | - | - | - | - | - | - | + | - | - | - | ? |
| **Macrodontia** | + | - | + | - | - | - | + | - | - | - | + | + |
| **Addiotional dental abnormalities** | wide upper central incisors | abnormality of his primary dentition | teeth malposition | - | - | - | - | - | - | - | - | - |
| **Cognitive impairment** | mild ID, language delay | learning difficulties | minor cognitive impairment with speech defect | developmental delay especially expressive language delay | moderate developmental delay (speech delay) | normal cognitive, speech delay | performace IQ 67, verbal IQ 81 | borderline ID (IQ 74) evaluated at the age of 20 years, speech was severely impaired (child), learning disabilities | global developmental delay | global developmental delay | ID | ID |
| **Autism spectrum disorder** | - | + | - | - | - | - | PDD-NOS | + | - | - | + | - |
| **Attention deficit disorder** | - | - | - | - | - | - | - | - | - | - | - | - |
| **Seizures** | - | - | - | - | - | - | - | + (refractory epilepsy) | - | possible seizure episode at 4 years of age | + (first at 1 year) | - |
| **Structural brain malformation** | ND | ND | moderate prenatal ventriculomegaly | - | ND | - | ND | cortical atrophia | slow myelinisation | possible undedevelopment of the inferior cerebellar vermis (CT scan of the brain at 4 years of age) | cortical atrophia | - |
| **Neuronal migration disorder** | ND | ND | ND | - | ND | - | ND | slight atypical morphology of hippocampus | - | - | - | - |
| **EEG abnormalities** | - | - | - | - | - | - | - | - | - | diffuse, very slow background activity, slow occipital rhythm, lateralization to the left occipital-posterior lobe temporal activity. No seizures. | - | - |
| **Ocular problems** | astigmatism | ND | myopia and astigmatism | ND | blepharitis and reduced cartilage in upper eyelids | glasses for astigmatism | ND | bilateral ptosis, right exotropia and failure of convergence | ptosis | intermittent esotropia, high hyperopia | ND | high myopia, ptosis left eye |
| **Hearing loss** | - | - | Conductive hearing loss (otitis) improved after tympanoplasty | - | Moderate sensorineural hearing loss | - | - | - |  | - | - | + |
| **Congenital heart defect** | VSD | VSD | - | Small muscular VSD, one membranous VSD with mildly dysplastic pulmonary valve. | ND | VSD | ND | - | VSD | hypoplasia of left ventricle, aortic annulus and aortic arch; clefted mitral valve | ND | ND |
| **Skeletal anomalies** | - | - | - | - | - | - | - (prox implantation of thumbs, hyperlax) | levoconvex scoliosis | + | - | + | - |
| **Hand/foot anomalies** | brachytelephalangy with brachydactyly of the second ray of the right hand and of the second, third, fourth and fifth rays of both feet | short fingers, small feet | fingers pads | single palmar crease right hand, proximally placed thumbs, rounded distal phalanx of thumbs and big toes | - | - | - | - | - | - | - | cutaneous sydnactyly II/II, III/IV and IV/V fingers |
| **Genital anomalies** | unilateral cryptorchidism (left) | - | - | - | undescended right testicle | - | - | undescended testes | - | - | - | - |
| **Hematologic disorder** | ND | - | ND | - | ND | neonatal trombopenia | ND | thrombocytopenia, low platelet and eosinophil counts, low urea, and low serum creatinine | thrombocytopenia | - | ND | - |
| **Delayed bone age** | + | - | + | + (age of 8 months at 19 months of age) | - | - | - | - | - | - | - | - |
| **Others** | - | cerebral palsy, difficult sleep | complex inner and middle ear abnormalities, premature puberty | asymmetrical skull shape, plagiocephaly, prominent sagittal suture, transitory leg hemihypertrophy | - | perinatal asphyxia with adrenal bleeds, resulting in hypocortisolism mild hypothyroidism | - | anosmia, slow and hypometric saccades, impaired upgaze with overactivity of frontalis | abnormality of the kidney, IUGR, increased susceptibility to fractures | - | stereotypies, bruxism, hand-biting, sleep disorders | automutilation, psoriasis, alopecia areata (head), cupid shape mouth, downturned corners mouth |

ND: not determined; ID: intellectual disability; PDD-NOS: pervasive developmental disorder not otherwise specified; VSD: ventricular septal defect; IUGR: intrauterin growth restriction.
