# Supplementary figures and images for: Haploinsufficiency for ANKRD11-flanking genes makes the difference between KBG and 16q24.3 microdeletion syndromes: 12 new cases
Source: Eur J Hum Genet. 2017 Apr 19;25(6):694–701. doi: 10.1038/ejhg.2017.49 (PMC5533198; doi:10.1038/ejhg.2017.49)

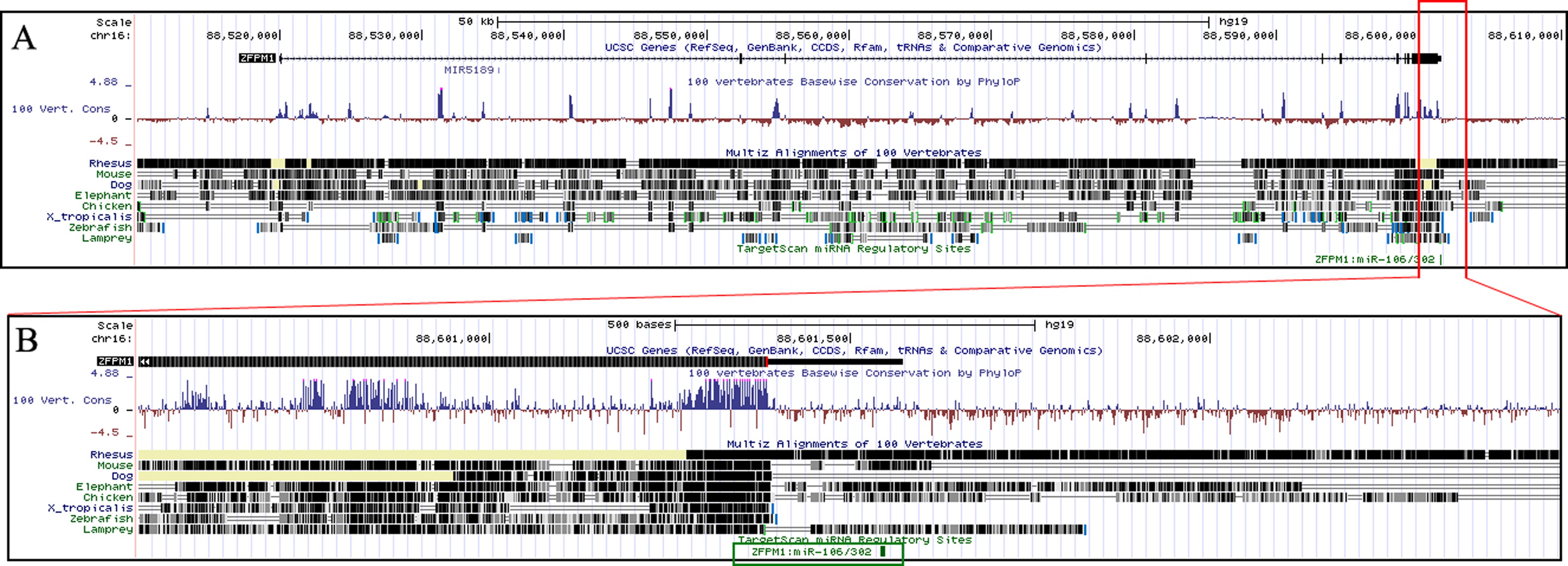

Supplement: Supplementary Figure S1 [file ejhg201749x3.tif]

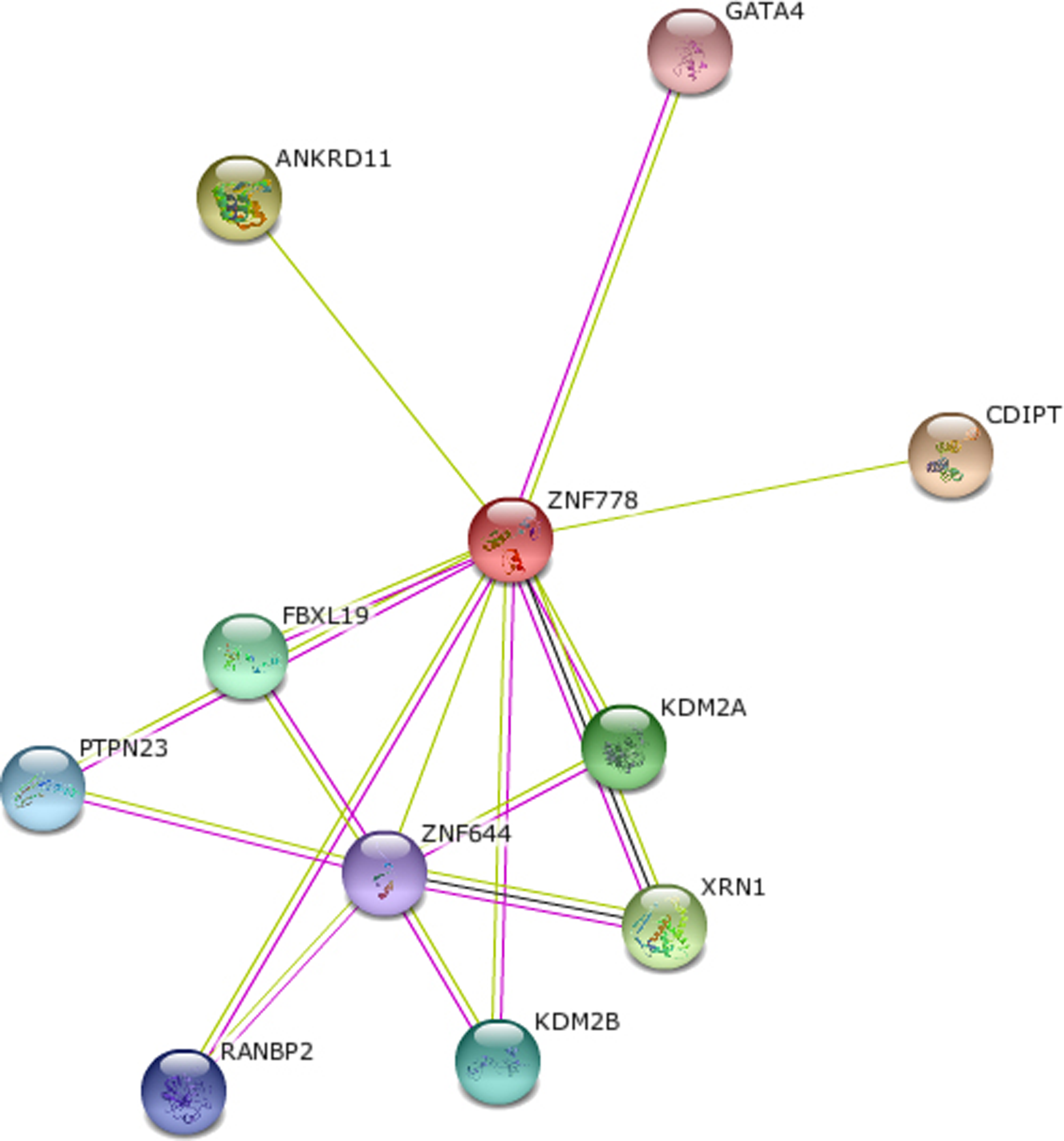

Supplement: Supplementary Figure S2 [file ejhg201749x4.tif]
